# Trimethylamine N-Oxide (TMAO) Acts as Inhibitor of Endothelial Nitric Oxide Synthase (eNOS) and Hampers NO Production and Acetylcholine-Mediated Vasorelaxation in Rat Aortas

**DOI:** 10.3390/antiox14050517

**Published:** 2025-04-25

**Authors:** Alma Martelli, Federico Abate, Michele Roggia, Giada Benedetti, Eugenio Caradonna, Vincenzo Calderone, Gian Carlo Tenore, Sandro Cosconati, Ettore Novellino, Mariano Stornaiuolo

**Affiliations:** 1 Department of Pharmacy, University of Pisa, Via Bonanno 6, 56120 Pisa, Italy; alma.martelli@unipi.it (A.M.); giada.benedetti@phd.unipi.it (G.B.); vincenzo.calderone@unipi.it (V.C.); 2 Department of Environmental, Biological and Pharmaceutical Sciences and Technologies DiSTABiF, University of Campania Luigi Vanvitelli, Via Vivaldi 43, 81100 Caserta, Italy; federico.abate@unicampania.it (F.A.); michele.roggia@unicampania.it (M.R.); sandro.cosconati@unicampania.it (S.C.); 3 Centro Diagnostico Italiano, Department of Clinical Laboratory, 20100 Milan, Italy; eugenio.caradonna@cdi.it; 4 Department of Pharmacy, School of Medicine and Surgery, University of Napoli Federico II, Via Domenico Montesano 49, 80131 Napoli, Italy; giancarlo.tenore@unina.it; 5 Department of Medicine and Surgery, Catholic University of the Sacred Heart, 00168 Rome, Italy

**Keywords:** Taurisolo^®^, trimethylamine N-oxide, endothelial nitric oxide synthase, acetylcholine-mediated vasorelaxation

## Abstract

Trimethylamine N-oxide (TMAO) is an endogenous osmolyte produced by enzymatic reactions starting in the human gut, where microbiota release trimethylamine (TMA) from foods, and ending in the liver, where TMA is oxidized to TMAO by flavin-containing monooxygenase 3 (FMO3). While physiological concentrations of TMAO help proteins preserve their folding, high levels of this metabolite are harmful and promote oxidative stress, inflammation, and atherosclerosis. In humans, elevated levels of circulating TMAO predispose individuals to cardiovascular diseases and chronic kidney disease and increase mortality risk, especially in the elderly. How TMAO exerts its negative effects has been only partially elucidated. In hypertensive rats, the eNOS substrate L-arginine and Taurisolo^®^, a nutraceutical endowed with TMAO-reducing activity, act synergistically to reduce arterial blood pressure. Here, we investigate the molecular mechanisms underpinning this synergism and prove that TMAO, the target of Taurisolo^®^, acts as direct inhibitor of endothelial nitric oxide synthase (eNOS) and competes with L-arginine at its catalytic site, ultimately inhibiting NO production and acetylcholine (Ach)-induced relaxation in murine aortas.

## 1. Introduction

In the last decade, the perception of the role played by the gut microbiota in human physiology has undergone a profound transformation. The trillions of bacteria colonizing the final section of the human gut can be no longer simply considered symbionts that, in exchange for nutrients and optimal environmental conditions, produce essential elements for our body (e.g., vitamin K2, short chain fatty acids, H_2_S, and methane). Compelling evidence reveals that the microbiota and its host should rather be considered as a meta-organism, where a sequence of interconnected, often bidirectional, chemical reactions occur to produce substrates and modulatory molecules necessary for the survival of the whole system [1,2,3]. The metabolites produced by these consecutive reactions exert profound endocrine effects on a multitude of the host’s physiological processes, impacting energy homeostasis, metabolism, neurological, and cardiovascular functions [4,5]. Interestingly, the fluctuations in these metabolites may also serve as important parameters for the early diagnosis of metabolic disorders and diseases [6].

Trimethylamine N-oxide (TMAO) is one of the metabolites produced by these sequential meta-organismal reactions. TMAO production begins in the intestine, where the microbiota (mainly *Clostridia, Shigella*, *Proteus*, *Aerobacter*, and *Eubacterium* species [7,8]) processes carnitine, choline, lecithin, betaine, and ergothioneine (all presenting trimethylamine (TMA) group) from dietary sources (e.g., red meat, eggs, saltwater fish, dairy products) [9]. Under intestinal anaerobic conditions [2,10], a bacterial glycyl-radical enzyme, encoded by prokaryotic *cutC* and *cutD* genes and endowed with choline-TMA lyase activity, converts choline into TMA. Similarly, l-carnitine is converted into TMA by the oxidoreductases encoded by *cntA*/*B* genes, and ergothioneine by the enzyme ergothionase [11]. The volatile TMA crosses the intestinal barrier, is transported by the portal vein, and reaches the liver, where it is oxidized to TMAO by flavin-containing monooxygenase 3 (FMO3) [12] and FMO1 [13], two microsomal cytochromes whose transcription is modulated by bile acids and upregulated by cholestasis. At physiological concentrations, TMAO acts as osmolyte and helps proteins maintain their correct folding, especially under conditions of osmotic stress. However, TMAO’s beneficial effect becomes detrimental when its blood or tissue concentration rises and its activity manifests mainly as an efficient pro-oxidant [10].

In murine models, elevated concentrations of TMAO lead to the oxidation of Low-Density-Lipoproteins (LDLs), oxidative stress, inflammation, cholesterol accumulation, and vessel thickening [10]. Dietary supplementation with TMAO promotes atherosclerosis in mice and rats, augmenting cholesterol accumulation in macrophages and foam cell formation [14]. In humans, high levels of TMAO are correlated with an increased risk of severe coronary atherosclerosis, hypertension [15,16], cardiovascular diseases (CVDs), and all-cause mortality [17,18,19,20]. Even in the absence of cardiovascular risk factors, TMAO remains a positive prognostic factor for mortality, especially in the elderly, with a 10 μM increment of TMAO corresponding to an almost 8% increase in the risk of all-cause mortality [21,22]. Recently, we have shown that intra-hospital variation in TMAO predicts future major adverse cardiovascular events after myocardial infarction [23]. Elevated circulating TMAO levels have also been linked to chronic kidney disease [24,25], insulin resistance [26], adiposity, impaired energy metabolism [16], pulmonary arterial hypertension [27], and peripheral artery disease [28].

Despite the evidence collected, the mechanism by which high levels of TMAO exert their plethora of negative effects remains incompletely understood. The small molecular dimensions of TMAO, along with its osmolyte nature, make this molecule virtually compatible with orthosteric and/or catalytic sites of different receptors and enzymes, especially those endowed with a high affinity for strong N^+^ → O^−^ dipoles. Identifying all TMAO targets is crucial for understanding its impact on human physiology and for developing strategies to counteract the deleterious effects of its increased concentration.

Recently, we have shown that Taurisolo^®^, a nutraceutical enriched in polyphenols capable of reducing circulating TMAO levels [28,29,30,31,32], presented, in vivo, a pharmacological synergism with L-arginine (the substrate of endothelial nitric oxide synthase (eNOS) [33]. In endothelial cells, eNOS is responsible for the synthesis of nitric oxide (NO) from L-arginine and plays a crucial role in regulating vascular function. Coupled and uncoupled states refer to the two different functional states of eNOS. In the coupled state, eNOS synthesizes NO and promotes vasorelaxation. In the absence of L-arginine (and/or of other cofactors), eNOS works in an uncoupled state and produces ROS, ultimately leading to oxidative damage, inflammation, and endothelial dysfunction. We have shown that L-arginine and Taurisolo^®^ synergistically reduced arterial blood pressure in spontaneously hypertensive rats as well as in a model of experimental dexamethasone-induced hypertension [33].

Here, we demonstrate, in vitro and ex vivo, that the synergism between Taurisolo^®^ and L-arginine can be explained by TMAO, the target of Taurisolo^®^, acting as an inhibitor of eNOS. Our findings shed light on an additional mechanism of TMAO action and underscore the importance of pharmacological treatments aimed at restoring physiological threshold levels of TMAO.

## 2. Materials and Methods

### 2.1. In Vitro Cell Culture and Treatment

Human umbilical vein endothelial cells, HUVECs (Life Technologies, Carlsbad, CA, USA), were cultured and seeded as previously reported [32]. Briefly, HUVECs (Life Technologies, Carlsbad, CA, USA) were cultured in Medium 131 (Life Technologies, Carlsbad, CA, USA) supplemented with 10% Fetal Bovine Serum, 100 μg/mL penicillin, 100 μg/mL streptomycin, 1% L-Glutamine, heparin 10 U/mL, epidermal growth factor (EGF, 10 ng/mL), and basic fibroblast growth factor (bFGF, 5 ng/mL) (Merck KGaA, Darmstadt, Germany). The culture was grown in 75 cm^2^ flasks (Nunc, Thermo Fisher Scientific, Waltham, MA, USA) at 37 °C in a humidified atmosphere of 5% CO_2_.

### 2.2. Fluorescent Detection of NO and ROS

HUVECs were seeded (5 × 10^3^/well) in a black 96-well plate (PerkinElmer, Waltham, MA, USA) in a final volume of 100 μL/well of growth media. When confluency reached 40%, cells were exposed to 10 μM Ach for 16 h, and when indicated, they were also introduced to various concentrations of TMAO (concentrations ranging from 10^−10^ to 10^−3^ M), 1 mM N(ω)-nitro-L-arginine methyl ester (L-NAME), 1–20 mM L-arginine, 0.1 μg/mL Taurisolo, 10 μM Sirtinol, and 10 μM Compound C, all dissolved in culture medium. ROS production in the HUVECs was measured with a fluorescent Dichlorofluoresceine diacetate probe (5 μM working solution, CM-H_2_DCFDA, # C6827, Invitrogen, Waltham, MA, USA) added to the culture 30 min before the end of the experiment. ROS production was quantified by measuring the relative fluorescent intensity (RFI) of CM-H_2_DCFDA (λ_exc_ = 485 nm and λ_ems_ = 535 nm) on an Envision 2105 plate reader (Perkin Elmer, Waltham, MA, USA). NO produced by HUVECs was measured with a fluorescent NO sensor DAF-FM Diacetate probe (5 μM working solution, Invitrogen, D23844) added to the culture 45 min before the end of the experiment. NO production was quantified by measuring the relative fluorescent intensity (RFI) of DAF-FM (λ_exc_ = 485 nm and λ_ems_ = 535 nm) on an Envision 2105 plate reader.

### 2.3. Measurement of L-Arginine Conversion to L-Citrulline in HUVECs Using Stable Isotope Labeling and LC-MS Analysis

HUVECs were plated on 75 cm^2^ flasks (Nunc, Thermo Fisher Scientific, Waltham, MA, USA) at 37 °C in a humidified atmosphere of 5% CO_2_. When confluency reached 80%, cells were supplemented with fresh medium containing 1 mM L-arginine and 0.1 mM deuterated L-arginine-2,3,3,4,4,5,5-d7. When indicated, cells were also treated with TMAO (50 μM), 0.1 mg/L Taurisolo, 10 μM Sirtinol, 10 μM Compound C, or their combination. Upon 3 h, the medium was removed and cells were washed two times in cold phosphate buffer saline and then extracted in 1 mL of pre-chilled methanol/water 1:1 solution, containing 10 nmol of internal deuterated standard; then, they were centrifuged at 10,000× *g* for 10 min at 4 °C. The resulting supernatants were collected and transferred into Eppendorf tubes. The supernatants were dried under nitrogen to be then reconstituted in 100 μL of ACN/H_2_O (70:30) (*v*/*v*) before HRMS analysis. Data were acquired on a SolariX XR 7T (Bruker Daltonics, Bremen, Germany). The instrument was tuned with a standard solution of sodium trifluoroacetate. Mass spectra were recorded in broadband mode in the range of 100–1500 *m*/*z*, with an ion accumulation of 20 ms, and with 32 scans using 2 million data points (2 M). Nebulizing (N_2_) and drying gases (air) were set at 1 and 4 mL/min, respectively, with a drying temperature of 200 °C. Five replicates of each injection were carried out. The instrument was controlled by Bruker FTMS Control, MS spectra were elaborated with Compass Data Analysis version 4.2 (Bruker, New York, NY, USA), and the identification of compounds based on accurate MS measurements was performed by Compound Crawler version 3.0 and Metaboscape 3.0 (Bruker, New York, NY, USA). Deuterated metabolite signals were normalized using internal deuterated standards. Comparisons and differences were analyzed for statistical significance by two-way Anova test and Bonferroni post-tests analysis.

### 2.4. Polyphenolic Content of Taurisolo^®^

Taurisolo^®^ is a nutraceutical polyphenolic supplement from Aglianico cultivar grapes, extracted with water at 50 °C and spray-dried with 5–15% maltodextrins as a support, resulting in a fine microencapsulated powder. The polyphenolic profile was evaluated using High-Performance Liquid Chromatography with a diode-array detector (HPLC-DAD, Jasco Inc., Easton, MD, USA), following the method described by Giusti et al. [34]. The composition of Taurisolo^®^ is detailed herein: Procyanidin B2 4135.7 ± 569.5 μg/g, Resveratrol 99.0 ± 7.12 μg/g, Catechin 22,047.3 ± 3638.8 μg/g, Epicatechin 15,521.0 ± 2503.9 μg/g, Quercetin-3-O-glucoside 3280.5 ± 451.1 μg/g, Quercetin 1620.7 ± 224.7 μg/g, Rutin 184.3 ± 33.6 μg/g, p-Coumaric acid 3468.4 ± 585.0 μg/g, Syringic acid 7174.3 ± 1069.0 μg/g, Gallic acid 4869.5 ± 871.6 μg/g, Caffeic acid 1436.7 ± 196.0 μg/g, Procyanidin B1 10,391.2 ± 746.7 μg/g, Procyanidin C1 6877.7 ± 564.3 μg/g, Kaempferol-3-O-glucoside 353.5 ± 3.32 μg/g, Ferulic acid 628.5 ± 54.5 μg/g, Epigallocatechin gallate 9180.6 ± 478.6 μg/g; for a total polyphenolic content of 544.8 ± 5.0 mg Gallic acid EQ/g [32]. Large-scale production was successfully carried out by MB-Med Company, located in Turin, Italy.

### 2.5. Animals Protocols and Ethical Statements

All the procedures involving animals were carried out following the guidelines of the European Community Council Directive 86–609 and in accordance with the Code of Ethics of the World Medical Association (Declaration of Helsinki, EU Directive 2010/63/EU for animal experiments). The experiments were carried out with the authorization of the Ethical Committee of the University of Pisa and the Italian Ministry of Health (authorization number 487/2020-PR). The animals were housed in cages with ad libitum access to water and food and maintained in controlled environmental conditions (humidity of 50% at 22 °C and with 12 h light/dark cycles). All possible measures were taken to reduce the number of animals used and to minimize their suffering. The animal studies were conducted in accordance with the ARRIVE guidelines [35].

### 2.6. Evaluation of the TMAO Effect on Rat Aorta Rings

Adult male normotensive Wistar rats (400–450 g, Envigo, Huntingdon, UK, RGD Cat# 2312511, RRID:RGD_2312511) were first anesthetized with an overdose of sodium thiopental (100 mg·kg^−1^ i.p., Pentothal MSD, Kenilworth, NJ, USA) and then killed by axillary exsanguination. The thoracic aorta was rapidly excised, freed of extraneous tissues, and cut into 5 mm-wide rings. These aortic rings were suspended under a preload of 2 g in organ baths containing 20 mL of Tyrode’s solution (NaCl, 136.8 mM; KCl, 2.95 mM; CaCl_2_·2H_2_O, 1.80 mM; MgSO_4_·7H_2_O, 1.05 mM; NaH_2_PO_4_·H_2_O, 0.41 mM; NaHCO_3_, 11.9 mM; and glucose, 5.5 mM), thermostated at 37 °C, and continuously gassed with Clioxicarb (95% O_2_ and 5% CO_2_) to mimic physiological conditions. An isometric transducer (Grass FTO3, Harvard Apparatus, Holliston, MA, USA) combined with a preamplifier (Buxco Electronics, Troy, NY, USA) and with software (BIOPAC Systems Inc., MP 100, LabChart 8.1.30, Goleta, CA, USA) recorded changes in tension. After an equilibration period of 30 min, the endothelial preservation was confirmed by the application of acetylcholine (Ach, Merck KGaA, Darmstadt, Germany) (10 μM) to KCl (25 mM)-precontracted vessel rings. For the endothelium-intact aortic rings, a relaxation ≥ 75% of the KCl-induced contraction was considered representative of an adequate presence of functional endothelium, whereas the rings with a relaxation < 70% were discarded. Each organ bath was washed and re-equilibrated with fresh Tyrode’s solution for 30 min.

In the first set of experiments, endothelial damage induced by TMAO was evaluated by comparing Ach-induced release curves. Different concentrations of TMAO (10, 20, 30 and 50 µM) or vehicle (Tyrode’s solution) were incubated in each organ bath for 1 h. Aortic rings were then contracted with KCl 25 mM and, after reaching a plateau, cumulative concentrations of Ach (10^−9^–10^−6^ M) were added. The Ach-induced vasorelaxant effect was expressed as a percentage (%) of the contractile tone induced by KCl.

Then, a second set of experiments was performed to assess the potential protection evoked by Taurisolo^®^ against the endothelial damage induced by TMAO at the selected concentration. Specifically, 2 h before the sacrifice, Wistar rats were given an i.p. injection of Taurisolo^®^ 20 mg/kg, Taurisolo^®^ 10 mg/kg, or the vehicle (physiological solution). The aorta was then excised, and aortic rings were suspended in organic baths to evaluate the endothelial preservation, as previously described. TMAO 30 or 50 µM were then incubated for 1 h to induce the endothelial damage, and therefore, cumulative Ach concentrations were added to the precontracted aortic rings. The Ach-induced vasorelaxant effect was expressed as a percentage (%) of the contractile tone induced by KCl 25 mM.

The impact of Taurisolo^®^ and TMAO on the smooth muscle was investigated in another set of experiments. The aortic rings of animals that received an i.p. injection of Taurisolo^®^ 20 mg/kg or vehicle solution 2 h before the sacrifice were suspended in organ baths after removing the endothelial layer with a needle. Endothelium-removed aortic rings were precontracted with 25 mM KCl and then with Ach (10 μM). An Ach-evoked relaxation < 10%, calculated as percentage of the KCl-induced contraction, was considered representative of an acceptable lack of the endothelial layer; conversely, the rings exhibiting a relaxation ≥ 10% were discarded. After a stabilization period of 30 min, 50 µM of TMAO or vehicle solution were incubated for 1 h. Then, a contraction was induced by 25 mM KCl administration and a cumulative concentration curve of SNP (10^−9^–10^−6^ M) was created for each aortic ring. The vasorelaxant effect promoted by SNP was expressed as a percentage of the maximum contraction induced by KCl 25 mM.

### 2.7. Statistical Analysis

For in vitro experiments, assays were run in triplicates. Data are presented as mean ± SD. Data points were fitted, and statistical analyses were performed using Prism 6 (GraphPad software). Comparisons between two groups were performed by using the *t*-test, analyzing each row individually, and not assuming consistent standard deviation. Statistical significance is expressed as a *p* value. Differences with *p* values > 0.05 were not considered statistically significant. For ex vivo experiments, vasorelaxant values expressed as % were fitted and statistically analyzed using Prism 8 (GraphPad software). The vasorelaxant response induced by Ach was expressed as a percentage (%) of the contractile effect induced by KCl. The potency index was expressed as pEC_50_, calculated as the negative logarithm of the molar concentration able to induce 50% of the maximal vasorelaxant effect. In contrast, the efficacy index, expressed as E_max_, was related to the maximum vasorelaxant effect obtained in that range of concentrations. Efficacy and potency parameters were expressed as mean ± SEM for each group. Concentration–response curves of aortas from different treatment groups were analyzed by two-way ANOVA followed by Bonferroni’s post-test. Differences between groups were considered significant at *p* < 0.05 and are marked with an asterisk (*).

### 2.8. In Silico Docking of TMAO in eNOS

The X-ray structure of eNOS (PDB code: 4D1O) [36] was sourced from the RCSB PDB database and underwent preliminary adjustments for docking purposes using the protein preparation wizard integrated into the Schrödinger suite [37,38]. TMAO’s structure was manually constructed using the 2D sketcher employed in Maestro. The molecule was subsequently exported as a PDB file and converted into PDBQT by making use of the AutoDock suite scripts [39]. The docking grid box was centered on the co-crystal structure, employing a box size of 40 40 40 (XYZ) with a spacing of 0.375 Å. Then, the receptor grid maps were calculated with the AutoGrid 4.0 [39] software, mapping the receptor interaction energies using every AutoDock atom type as a probe. For the docking calculations attained through AD4-GPU [40], the Lamarckian Genetic Algorithm (LGA) was employed, encompassing a total of 100 LGA runs. All other settings were maintained at their default values. The docking results were subsequently grouped based on the RMSD criterion, whereby solutions differing by less than 3.0 Å were considered part of the same cluster. The ranking of these clusters was determined based on the calculated free energy of binding (ΔG_AD4_).

## 3. Results

We have already shown that the TMAO-reducing activity of Taurisolo^®^ might be explained by a bimodal mechanism of action [41]. Thanks to its antioxidant activity, Taurisolo^®^ lowers humans’ circulating TMAO levels by reducing the pro-oxidant molecule back to TMA [30], its reduced form. Moreover, Taurisolo^®^ stimulates intracellular antioxidant defense [42], promotes mitochondrial metabolism [43], and activates AMPK as well as the Sirtuin pathway [32]. Recently, we showed that Taurisolo^®^ presented a pharmacological synergism with the eNOS substrate L-arginine in vivo. L-arginine and Taurisolo^®^ synergistically reduce arterial blood pressure in murine models of hypertension [33].

In this work, we propose that the observed synergistic effect between Taurisolo^®^ and L-arginine could be explained by TMAO’s role as a direct inhibitor of endothelial nitric oxide synthase (eNOS). We hypothesize that Taurisolo^®^, by reducing TMAO levels, indirectly enhances eNOS activity, which is further boosted by L-arginine supplementation. This mechanism could account for the synergistic improvement observed when these two compounds are used in combination.

### 3.1. TMAO Docks in the L-Arginine Binding Site of eNOS

**Figure 1:**
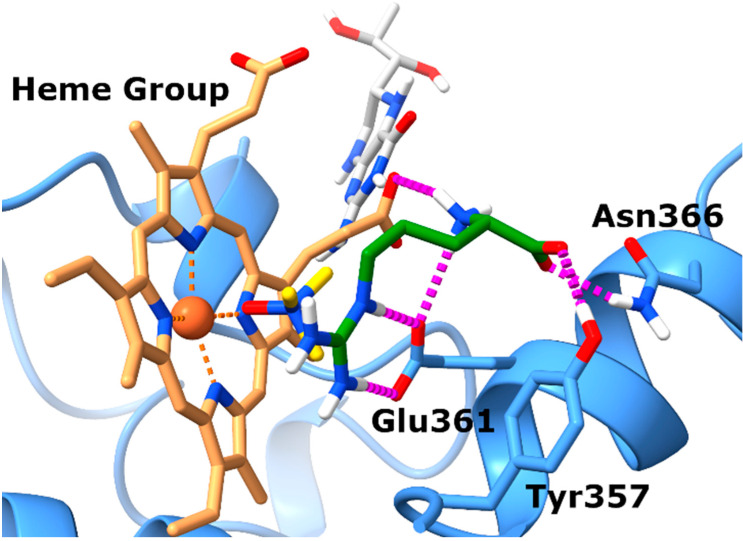
TMAO docks in the L-arginine binding site of eNOS. Binding mode of TMAO (docked) and L-arginine (experimental) into the X-ray eNOS (PDB ID: 4D1O) structures. The protein is represented as blue ribbons and sticks. Electrostatic interactions are represented as dashed magenta lines, the ligands are represented as yellow (TMAO) and green (L-arginine) sticks, respectively, while the Heme group and the BH4 cofactor are represented as brown and gray sticks, respectively.

Prior to experimental validation, we conducted in silico studies to assess whether TMAO could effectively bind to the eNOS orthosteric site, potentially hindering the interaction with its natural substrate L-arginine. This computational analysis served as a preliminary step to exploring the plausibility of our hypothesis. Specifically, docking experiments were completed by employing the X-ray structure of eNOS (PDB code: 4D1O) in complex with its endogenous substrate L-arginine. The docking experiments (Figure 1) highlighted a single well-defined binding pose for TMAO, closely resembling that of the co-crystal. In particular, the N-oxide group of TMAO points towards the heme group of the eNOS protein establishing proficient monodentate chelation of the Fe^2+^ ion. Also, the positively charged nitrogen of TMAO might establish cation–π interactions with the aromatic moieties of the prosthetic group.

Analysis of the position of the co-crystalized L-arginine evidences that the guanidinium group faces the tetrapyrrole ring and contacts the enzyme Glu361 residue via ionic interactions. Regarding the backbone of L-arginine, it also establishes favorable contacts with nearby amino acids. In particular, the carboxylate forms charge-reinforced H-bonds with both Tyr357 and Asn366 side chains, while the backbone NH_3_^+^ group forms a salt bridge with the propionate group of heme.

In summary, the docking results obtained for TMAO allow for postulation that it shares the same binding site with L-arginine, suggesting that it might compete with the endogenous substrate to bind to the catalytic domain of eNOS, preventing the synthesis of nitric oxide (NO).

### 3.2. TMAO Competes with L-Arginine at eNOS Catalytic Site and Inhibits NO Production

**Figure 2:**
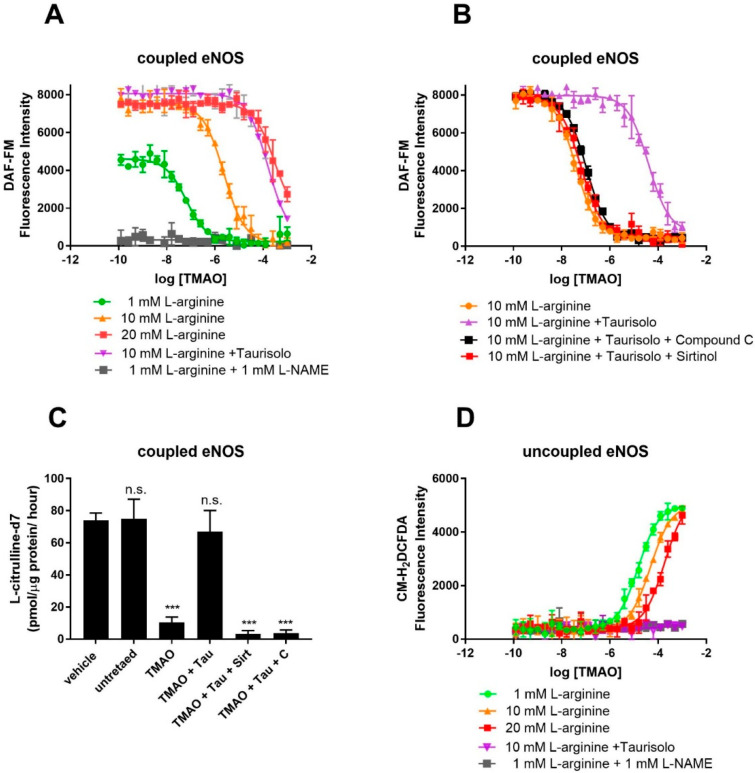
TMAO interferes with eNOS’s NO-synthesizing activity and stimulates eNOS-dependent ROS production in cultured HUVECs. Detection of endogenous NO (**A**) (measured by DAF-FM fluorescence) in HUVECs in the presence of the indicated concentration of TMAO (x-axis), 10 μM Ach and 1, 10, 20 mM L-arginine, in the presence or absence of 1 mM L-NAME. (**B**) Inhibition of NO production by TMAO and its rescue by co-incubation with 100 mg/L Taurisolo, in the presence or absence of AMPK (Compound C 10 μM) and Sirtuin inhibitor (Sirtinol 10 μM). (**C**) Rate of conversion of deuterated L-arginine-2,3,3,4,4,5,5-d7 into L-Citrulline-2,3,3,4,4,5,5-d7 in HUVECs in the presence of 20 μM TMAO, 100 mg/L Taurisolo, 10 μM Compound C, or 10 μM Sirtinol. (**D**) Detection of endogenous ROS measured in HUVECs in the presence of the indicated concentration of TMAO (x-axis), 10 μM Ach and 1, 10, 20 mM L-arginine, in the presence or absence of 1 mM L-NAME. Data are presented as a mean with error bars and represent the average ± SD of 3 independent experiments (*** *p* value ≤ 0.001, n.s. means “not significant”). For **A**, **C**, and **D**, data were fitted with a dose–response curve to identify the EC50 of ROS stimulation (**D**) and NO inhibition (**A**,**C**).

To confirm competition between TMAO and L-arginine at the eNOS binding site, eNOS activity was monitored in HUVECs using the fluorescent NO-probe DAF-FM and by measuring the conversion of L-arginine into L-citrulline by HPLC-MS. For the fluorescent assay, NO production was achieved by supplementing culture media with Ach (10 μM) and L-arginine (1 mM). As shown by the fluorescent emission of the NO probe, in the absence of TMAO, HUVECs efficiently produced NO (Figure 2A). NO production is indeed eNOS-dependent, as proven by the eNOS inhibitor L-NAME, whose presence strongly abolishes NO production. Exposure to TMAO [concentration ranging from 10^−10^ to 10^−3^ M] for 60 min abolishes NO production through a dose-dependent inhibition, with a logEC_50_ of −5.6 ± 0.05 (EC_50_ = 2.5 ± 0.3 µM). Supplementation with an excess of L-arginine (20 mM) rescued NO production, indeed suggesting that TMAO and L-arginine might compete for the same binding site. As expected, Taurisolo^®^ abolished TMAO’s inhibitory activity on eNOS. As already shown [32], Taurisolo’s mechanism of action in endothelial cells and tissues involves AMPK and Sirtuin. Indeed, eNOS activity impaired by TMAO was not re-established when Taurisolo was supplemented in the presence of the AMPK inhibitor Compound C or the Sirtuin inhibitor Sirtinol (Figure 2B). Finally, to confirm that TMAO was indeed affecting eNOS activity rather than NO availability, we measured the rate of L-arginine’s conversion to L-citrulline by using stable isotope labeling and mass spectrometry. The results confirm that TMAO inhibits L-arginine’s conversion to L-citrulline and that eNOS activity can be restored by Taurisolo through an AMPK- and Sirtuin-dependent mechanism (Figure 2C).

### 3.3. TMAO Is an Inhibitor of eNOS and Promotes eNOS-Dependent ROS Production

Under conditions of L-arginine depletion, eNOS works in its uncoupled state and produces ROS. Uncoupled eNOS activity in HUVECs was monitored by measuring ROS production with the ROS Dichlorofluoresceine diacetate probe. As shown by Figure 2D, in the absence of TMAO, HUVECs produce a minimal amount of ROS (Figure 2D). Exposure to TMAO [ranging concentration from 10^−10^ to 10^−3^ M] for 1 h promotes ROS production through a dose-dependent manner of inhibition, with a logEC_50_ of −4.8 ± 0.1 (EC_50_ = 15.8 ± 0.1 µM). ROS production is indeed eNOS-dependent, as proven by the eNOS inhibitor L-NAME, whose presence strongly abolishes ROS production. Similarly, supplementation with an excess of L-arginine (20 mM) abolished ROS production, again pointing toward TMAO and L-arginine competing for the same binding site.

According to our in silico and in vitro data, TMAO binds eNOS at the same binding site as L-arginine and induces a pharmacological response opposite to that of the endogenous ligand. TMAO might thus be classified as an eNOS inhibitor.

### 3.4. Ex Vivo Inhibitory Activity of TMAO on Ach-Induced Vasorelaxation of Rat Aortas

**Figure 3:**
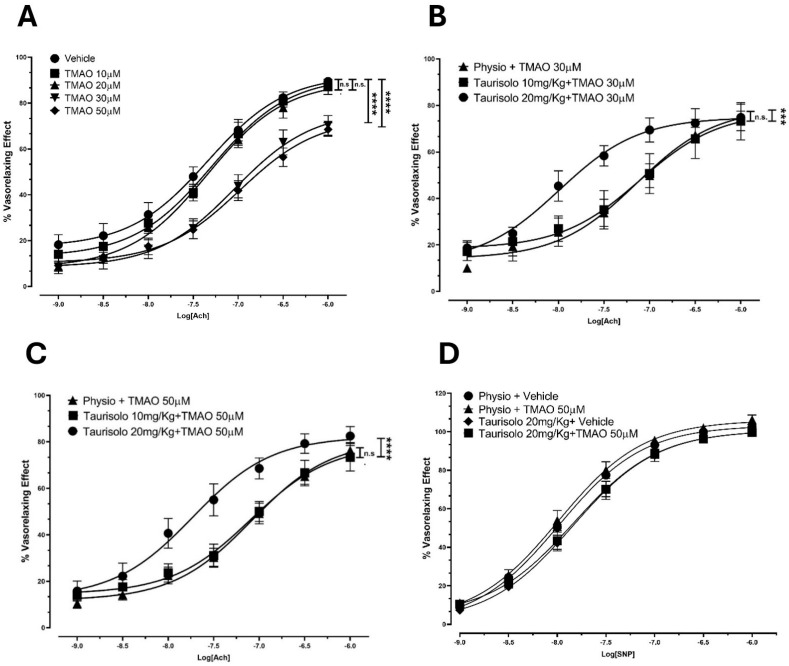
Endothelial impairment induced by TMAO and ability of Taurisolo^®^ 20 and 50 mg/kg to significantly prevent TMAO-induced reduction in Ach-evoked vasorelaxant curve response at endothelial level. (**A**) Endothelial impairment induced by TMAO. Concentration–response curve induced by Ach on pre-contracted endothelium-intact rat aortic rings. Incubation with 30 and 50 µM TMAO induced a significant reduction in the Ach-induced vasorelaxant response, compared to the vehicle group. The vertical bars indicate the SEM. The asterisks indicate a significant difference from the vehicle curve obtained on endothelium-intact aortic rings (**** *p* < 0.0001); n.s. means “not significant”. (**B**) Concentration–response curve induced by Ach on pre-contracted aortic rings (from rats that received Taurisolo^®^ 10 mg/kg, 20 mg/kg, or physiological solution) incubated with 30 µM TMAO. Taurisolo^®^ 20 mg/kg showed the ability to significantly prevent the 30 µM TMAO-induced reduction in the Ach-evoked vasorelaxant curve response. The vertical bars indicate the SEM. The asterisks indicate a significant difference from the response obtained on endothelium-intact aortic rings from the group treated with physiological solution (*** *p* < 0.001); n.s. means “not significant”. (**C**) Concentration–response curve induced by Ach on pre-contracted aortic rings (from rats that received Taurisolo^®^ 10 mg/kg, 20 mg/kg, or physiological solution) incubated with 50 µM TMAO. Taurisolo^®^ 20 mg/kg significantly increased the Ach-induced vasorelaxant curve response compared to the physiological solution group (physio). The vertical bars indicate the SEM. The asterisks indicate a significant difference from the physio curve obtained for endothelium-intact aortic rings (**** *p* < 0.0001); n.s. means “not significant”. (**D**) Concentration–response curve induced by SNP on endothelium-denuded, pre-contracted aortic rings (from rats that received Taurisolo^®^ 20 mg/kg or physiological solution) incubated with 50 µM TMAO or vehicle solution. Physiological solution: physio. No differences were recorded in vasorelaxant effect of endothelium-denuded aortic rings (derived from rats injected with physiological solution or Taurisolo^®^ 20 mg/Kg) treated with vehicle or TMAO 50 µM.

To confirm TMAO’s inhibitory activity on eNOS in an ex vivo platform, we assessed Ach-induced vasorelaxant responses in rat aortas upon incubation with TMAO (10, 20, 30, 50 µM) or vehicle solution. TMAO incubation induced a reduction in the Ach-induced vasorelaxant response in endothelium-intact rat aortic rings pre-contracted with KCl, compared to the vehicle group. In particular (Figure 3A), in the vehicle-pretreated aortic rings, Ach induced a sustained vasorelaxation, with an E_max_ of 89.5 ± 1.9% and a potency value (pEC_50_) of 7.30 ± 0.10. Pretreatment with TMAO 10 and 20 µM did not show any significant variation in Ach-evoked vasorelaxant responses compared to the vehicle conditions. Pretreatment with TMAO 30 and 50 µM resulted in a significant decrease in both the efficacy (E_max_ of 70.3 ± 2.9% and 68.5 ± 4.3%, respectively) and the potency (pEC_50_: 7.00 ± 0.15 and 6.90 ± 0.23, respectively).

We thus tested if the TMAO inhibitor Taurisolo^®^ could restore Ach-evoked vasorelaxation. After the selection of 30 and 50 µM TMAO concentrations as the most appropriate to induce a significant endothelial impairment, the protection provided by the administration of Taurisolo^®^ was assessed. We have already shown that Taurisolo^®^ promotes vasorelaxation thanks to its antioxidant power and its ability to modulate the master regulator AMPK. Here, Taurisolo^®^ showed the ability to prevent the TMAO-induced reduction in the Ach-evoked vasorelaxant curve response, demonstrating a protective effect against endothelial dysfunction. Indeed, after incubation with TMAO 30 µM (Figure 3B), aortic rings from animals that received an injection of physiological solution showed a vasorelaxant response characterized by an E_max_ of 75.0 ± 2.5 and a pEC_50_ of 7.1 ± 0.2. Animals pre-treated with an injection of Taurisolo^®^ 10 mg/kg showed a similar vasorelaxant curve (E_max_ 73.2 ± 3.3, pEC_50_ 7.10 ± 0.30). However, a Taurisolo^®^ dosage of 20 mg/kg enhanced Ach-induced vasorelaxant response, reaching a final comparable efficacy (E_max_ 74.8 ± 5.7) but inducing 50% of the maximal vasorelaxant effect with a concentration 10 times lower (pEC_50_ 8.0 ± 0.2) than the vehicle addition. The same trend was observed in rings incubated with TMAO 50 µM (Figure 3C). Aortic rings from animals treated with physiological solution, showed an impaired vasorelaxation after incubation with TMAO 50 µM, resulting in an E_max_ value of 76.3 ± 3.3 with a potency value (pEC_50_) of 7.10 ± 0.10. As already observed, pre-treatment with Taurisolo^®^ 10 mg/kg did not significantly affect the vasorelaxant response (E_max_ 73.3 ± 5.9, pEC_50_ 7.1 ± 0.2), whereas treatment with Taurisolo^®^ 20 mg/kg showed a significant recovery of the vasorelaxant response (E_max_ 82.5 ± 4.1, pEC_50_ 7.70 ± 0.20).

TMAO does not affect smooth muscle. Indeed, no differences were observed in the SNP-induced vasorelaxant response of denuded aortic rings from animals injected with physiological solution or Taurisolo^®^ 20 mg/kg and pre-incubated with vehicle or TMAO 50 µM (Figure 3D). Aortic rings from animals treated with physiological solution and pre-incubated with vehicle solution (Emax 103.30 ± 0.9, pEC_50_ 8.0 ± 0.03) showed an SNP-induced vasorelaxation comparable with the results obtained after incubation with TMAO 50 µM (Emax value of 106.0 ± 2.1, pEC_50_ of 8.00 ± 0.06). Similar effects were observed in denuded aortic rings from animals previously injected with Taurisolo^®^ and incubated with vehicle (Emax 100.70 ± 1.8, pEC_50_ 7.8 ± 0.06) or TMAO 50 µM (Emax 100.70 ± 1.9, pEC_50_ 7.8 ± 0.06).

These results confirm that the previously observed TMAO-induced reduction in vasorelaxation did not affect the smooth muscle but was completely attributable to TMAO-evoked endothelial damage, and the protective effect of Taurisolo^®^ may be due to the preservation of endothelial function.

## 4. Discussion

CVDs are among the main causes of mortality worldwide [44], and atherosclerosis and hypertension represent two important CVD risk factors. In recent years, the link between dysbiosis, gut microbiota, microbiota-derived metabolites, and CVDs has been supported by clinical data and metanalysis. One of these metabolites, TMA, together with its oxidized form, TMAO, links nutrition, life habits, and microbiota to humans’ risk of developing CVDs [17,18,19,20]. Pharmacological treatments aimed at restoring physiological threshold levels of TMAO need to be identified.

Antioxidants are promising candidates to restore TMAO physiological levels or counteract its activity. Resveratrol, highly enriched in nutraceuticals, was shown by other authors to reduce TMAO levels and atherosclerosis in a murine model when used as a pure molecule [45]. In our clinical trials, Taurisolo^®^ reduced serum levels of TMAO, oxLDL, ROS, and other biomarkers associated with atherosclerosis [30,31]. Among other antioxidants, vitamin D supplementation over three months has been associated with a reduction in circulating TMAO levels, alongside an increase in NO [46].

Acute as well as long-term supplementation with Taurisolo^®^, a TMAO inhibitor, significantly increased flow-mediated dilation in healthy subjects [32]. In spontaneously hypertensive and dexamethasone-induced hypertensive rats, supplementation with L-arginine and Taurisolo^®^ synergistically reduced arterial blood pressure [33]. Here, we investigated the molecular mechanisms underpinning this synergism by proving that TMAO, the target of Taurisolo^®^, competes with L-arginine at the eNOS catalytic site and inhibits coupled eNOS activity and NO production. Coupled eNOS is associated with cardiovascular protection, as NO production promotes vasorelaxation and inhibits atherosclerosis. The synergism between L-arginine and Taurisolo^®^ might represent a strategy to counteract ROS production by eNOS and support the healthy functioning of the cardiovascular system.

Our in vitro results point towards competition between TMAO, L-arginine, and L-NAME for the same binding site. TMAO binding to eNOS induces a pharmacological response opposite to that of L-arginine, stabilizing eNOS’s uncoupled state and triggering ROS production. While we cannot exclude TMAO’s potential to also occupy other allosteric sites, the data presented here point toward TMAO exerting its eNOS inhibitory activity by acting as a direct inhibitor.

The dysregulation of coupled eNOS promoted by TMAO could explain the correlation between TMAO blood levels, hypertension, and the progression of cardiovascular diseases. Interestingly, the EC_50_ of inhibition we measured in vitro and ex vivo falls in the range of concentrations measured in humans, suggesting that eNOS inhibition by TMAO could occur in vivo. Our data are in line with previous reports evidencing TMAO regulating vasorelaxation and controlling blood pressure depending on its concentration. Here, we prove, both in vitro and ex vivo, that physiological concentrations of TMAO do not affect eNOS activity. Similarly, Jomard et al. showed how the preincubation of human endothelial aortic cells with 10^−6^ M TMAO did not affect NO production nor hamper ACh-induced rat aorta relaxation ex vivo [47]. Differently, elevated levels of TMAO worsen the outcomes of pressure overload-induced heart failure [19], exacerbate the hypertensive effect of angiotensin II [48], and promote atherosclerotic development [45,49]. Hypertension in diabetic patients has been linked to the hyperexpression of hepatic FMO3, the enzyme responsible for TMAO production, as a consequence of cholestasis associated with the disease [50]. Brunt et al. showed that TMAO administration induces an aging-like deterioration of endothelial function through superoxide-associated oxidative stress and an impairment of NO bioavailability [51]. Recently, Saaoud et al. showed that TMA can also be oxidized to TMAO in the aorta by endothelial FMO3 [52].

Despite not hypothesizing a direct binding between TMAO and eNOS, Sun et al. have shown that high levels of TMAO inhibit eNOS activity and NO bioavailability, although the authors did not confirm that TMAO impairs NO production. Interestingly, the authors show that TMAO-mediated effects are significantly reversed by treatment with the antioxidant N-acetylcysteine [49], which, in virtue of its reducing potential, could indeed convert TMAO to TMA, like Taurisolo^®^. According to Sun, eNOS inhibition by TMAO causes a release of the proinflammatory cytokines IL-1β and IL-18 and involves NLRP3, a component of the inflammasome [49], suggesting that inflammation is likely a mechanism involved in eNOS inhibition by TMAO. However, the involvement of inflammation in TMAO’s inhibitory effect on eNOS is controversial. Matsumoto et al. confirmed that TMAO is a causal factor in the development of peripheral artery disease and that the molecule impairs ACh-induced and endothelium-derived hyperpolarizing factor-mediated relaxation in femoral arteries. However, TMAO’s inhibitory activity was not abolished by indomethacin (a cyclooxygenase inhibitor), suggesting that TMAO’s inhibitory effect on eNOS does not involve its proinflammatory activity [53]. However, inflammatory processes are not solely a result of COX-derived prostanoids, and thus further experiments are required to clarify the role of TMAO in inflammation.

While shedding light on an additional mechanism underpinning TMAO toxicity, this study investigates the mechanism causing synergism between L-arginine and Taurisolo^®,^ presenting this combination as an eligible target mechanism for treatment aimed at restoring physiological threshold levels of TMAO and contributing to cardiovascular homeostasis [30,31,32,33,54]. We cannot, however, exclude the possibility that TMAO could also exert other indirect effects on vascularization. Syu et al. have shown that TMAO affects neovascularization and tissue regeneration by inhibiting human endothelial progenitor cells’ migration and differentiation into endothelial cells. The authors propose that the inhibitory effect of TMAO on these cells involves, among others, inactivation of the AKT/eNOS pathway [55]. Querio et al., while confirming that TMAO interferes with physiological vasorelaxation by inhibiting NO release, propose the modulation of eNOS phosphorylation at Ser1179 and the involvement of ATP-induced intracellular calcium and mitochondrial health as consequences of prolonged treatment with TMAO [56].

## 5. Conclusions

Here, we provide compelling evidence that TMAO directly inhibits eNOS by competing with its physiological substrate, L-arginine, at the catalytic binding site. This interference results in a significant reduction in NO production, thereby impairing acetylcholine-mediated vasorelaxation in both in vitro and ex vivo models. The molecular docking analysis supports the hypothesis that TMAO occupies the same binding site as L-arginine within the eNOS structure, which is further validated by functional assays demonstrating a dose-dependent inhibition of NO synthesis and enhanced ROS generation. Importantly, the nutraceutical Taurisolo^®^, through its antioxidant and TMAO-reducing activities, restores eNOS functionality and endothelial responsiveness, particularly when used in combination with L-arginine. These findings elucidate a mechanistic basis for the adverse cardiovascular effects associated with elevated TMAO levels and propose a novel therapeutic strategy aimed at re-establishing vascular homeostasis through the targeted modulation of eNOS activity.

## Data Availability

The data used to support the findings of this study are included within the article.
